# Validation of screening tools for common mental health disorders in the methadone maintenance population in Hanoi, Vietnam

**DOI:** 10.1186/s12888-021-03493-8

**Published:** 2021-10-05

**Authors:** Anisa Y. Mughal, Melissa A. Stockton, Quynh Bui, Vivian Go, Tran Viet Ha, Brian W. Pence, Bradley N. Gaynes

**Affiliations:** 1 The University of Pittsburgh School of Medicine, 3550 Terrace Street, Pittsburgh, PA 15213 USA; 2 Epidemiology Department, University of North Carolina at Chapel Hill Gillings School of Global Public Health, 135 Dauer Dr, Chapel Hill, NC 27599 USA; 3 The UNC Vietnam Office, Yen Hoa Health Clinic, Lot E2, Duong Dinh Nghe Street, Yen Hoa Ward, Cau Giay District, Hanoi, Vietnam; 4 Department of Health Behavior, University of North Carolina at Chapel Hill Gillings School of Global Public Health, 135 Dauer Dr, Chapel Hill, NC 27599 USA; 5 Department of Psychiatry, University of North Carolina at Chapel Hill School of Medicine, 333 S Columbia St, Chapel Hill, NC 27516 USA

**Keywords:** Depression, Anxiety, Post-traumatic stress disorder, Validation, Methadone, Drug use

## Abstract

**Background:**

Common mental health disorders (CMDs), including depression, anxiety and post-traumatic stress disorder (PTSD) may worsen both HIV and drug use outcomes, yet feasible tools to accurately identify CMDs have received limited study in this population. We aimed to validate the Patient Health Questionnaire (PHQ-9), Generalized Anxiety Disorder screen (GAD-7) and Primary Care PTSD screen for DSM-5 (PC-PTSD-5) in a methadone maintenance therapy (MMT) patient population in Hanoi, Vietnam.

**Methods:**

We conducted a cross-sectional survey. The PHQ-9, GAD-7, and PC-PTSD-5 were administered to MMT patients. A blinded interviewer administered the Mini-International Neuropsychiatric Interview (MINI) as the reference gold standard. Total scores of each tool were compared with the MINI diagnoses using a receiver operating characteristic curves, and we identified the optimal respective cut-off scores using the Youden’s Index.

**Results:**

We enrolled 400 MMT patients. Approximately 99.3% were male (*n* = 397) and 21.8% (*n* = 87) were HIV positive. The prevalence of major depressive disorder, generalized anxiety disorder and PTSD, respectively, was 10.5, 4 and 2%. Optimal cut-off scores for the PHQ-9, GAD-7 and PC-PTSD were ≥ 5, ≥3, and ≥ 4 with a sensitivity/specificity of 95.2%/91.9, 93.8%/87.5, and 62.5%/95.2%.

**Conclusions:**

The prevalence of CMDs in the MMT population was lower than expected. A lower cut-off score may be considered when screening for CMDs in this population. Further research should investigate the validity of somatic symptom-based screening tools among other drug-using or MMT populations.

## Background

Common mental health disorders (CMDs) – including depression, anxiety and post-traumatic stress disorder (PTSD) – are leading contributors to disability adjusted life years (DALYs) globally [1, 2] and are highlight prevalent among patients with opioid use disorder and those on methadone maintenance therapy (MMT) [3, 4]. CMDs in low and middle income countries (LMICs) such as Vietnam are less frequently identified and treated than in high-income countries (HICs) despite an increased share in burden of disease [2]. Further, providers often only identify severe mental disorders when confronted with visible psychotic or manic symptoms; less severe disorders are often overlooked, as CMDs such as depression may not be considered a disorder but rather a state of simply “thinking too much” [5]. Lack of funding, stigma surrounding mental health, mental healthcare accessibility, limited psychiatric human resources and infrastructure, and few validated screening tools have hindered the study of CMDs in LMICs such as Vietnam [6–8].

Only a few CMD screening tools for depression and anxiety have been validated in Vietnam. These tools include the DASS-21 (Depression, Anxiety and Stress Scale), Zung SAS (Zung Self-Rating Anxiety Scale), GHQ-12 (General Health Questionnaire), SRQ-20 (Self-Reporting Questionnaire) and EPDS (Edinburgh Postnatal Depression Scale), which were validated against the Structured Clinical Interview for DSM-5 (SCID) or the Composite International Diagnostic Interview (CIDI) [9–11]. Additionally, another study examined the psychometric properties of the DASS-21 among adolescents [12]. These studies did not always include a reference gold standard and were restricted to specific sub-populations, such as women, perinatal women and their male partners, and adolescents in the northern regions [9–12]. While a wide range of CMD screening tools have been designed, developed and tested in HICs, many, particularly for PTSD, have not been locally validated in LMICs such as Vietnam [5]. Local validation ensures the accuracy of the screening tool and can facilitate improved detection and diagnosis of mental health disorders. This confirmation is especially important for populations at high risk of developing CMDs, such as patients with opioid use disorder or on MMT.

In Vietnam, over 220,000 people are estimated to inject drugs, and an estimated 51,000 have enrolled in MMT, often at clinics in their communities [13, 14]. These patients with opioid use disorder and those on MMT have a high risk of having an undetected CMD [15]. CMDs within the MMT population are up to 10 times more prevalent than in the general population [15], and still two to three times higher than in substance users not on methadone [16]. At MMT enrollment, patients are not routinely screened for CMDs. As such, an adequate record detailing CMD prevalence while in treatment thereafter are virtually nonexistent. Further, very few studies have estimated the prevalence of CMDs among populations with opioid use disorder or on MMT in Vietnam, and none have estimated the prevalence of PTSD [8, 17–19]. CMDs can hamper MMT compliance [20] which can exacerbate challenges to continued engagement in MMT. This challenge suggests a need for screening and treatment of CMDs within MMT populations. However, to our knowledge, no studies have focused on validating screening tools for CMDs in an urban environment among the MMT population, almost 25% of whom are living with HIV (PLWH) and at an additional high risk for CMDs.

In order to address this research gap, we aimed to validate screening tools for depression, anxiety and PTSD in a MMT population at an urban clinic in Hanoi to ensure the tools’ accuracy when compared to a reference gold standard.

## Methods

### Screening tools

The Patient Health Questionnaire (PHQ-9) is a nine-item questionnaire to screen for depression [21]. It is scored using a Likert scale according to duration of symptoms such as low mood, guilt, lack of appetite, changes in sleep or thoughts or hurting oneself. The Generalized Anxiety Disorder scale (GAD-7) is a seven-item questionnaire that is used to screen for generalized anxiety disorder [22]. It is scored using a Likert scale according to duration of symptoms including feelings of nervousness, anxiousness or trouble relaxing. The Primary Care PTSD Screen for DSM-5 (PC-PTSD-5) is a five item yes/no screening tool to detect PTSD regarding symptoms related to a traumatic event causing trouble sleeping, flashbacks or feelings of numbness or detachment [23]. The Mini International Neuropsychiatric Interview (MINI) is a validated structured clinical interview consistent with the DSM-5 divided into sections for different disorders, such as major depressive disorder, psychotic disorders, eating disorders and other mood disorders [24].

The Vietnamese versions of the Patient Health Questionnaire (PHQ-9) and Generalized Anxiety Disorder screen (GAD-7) were taken from previous studies [25–28], and the Primary Care PTSD Screen for DSM-5 (PC-PTSD-5) was translated into Vietnamese by a bilingual clinical staff member who had translated for previous studies. Translation of the MINI for DSM-5 into Vietnamese was conducted by a bilingual clinical staff member following four steps. First, the depression, anxiety and PTSD sections of the MINI for DSM-5 were translated into Vietnamese. Second, the translation was independently compared to an existing validated translation of the MINI for the DSM-4 used in Hai Phong [17]. Third, the translation of the MINI for DSM-5 was reviewed for cultural applicability and comprehensibility by a group of Vietnamese research staff including physicians and research assistants with previous translation experience. Fourth, the two interviewers for the study conducted practice interviews to determine feasibility and acceptability of the translation that was overseen by a psychiatrist.

### Ethical consideration/approval

All participants gave a written informed consent in agreement with the Helsinki Declaration. The study was approved by the Nam Tu Liem District Health Center Ethical Review Board in Vietnam and by The UNC Office of Human Research Ethics/Institutional Review Board (OHRE/IRB) in the United States.

### Study procedures

A consecutive sampling technique was used to recruit 420 adult patients enrolled in an urban methadone maintenance therapy clinic. Patients were approached by MMT staff for interest in participation. As requested and approved by the University of North Carolina at Chapel Hill IRB, we verbally consented patients to maximize confidentiality.

Interviews were conducted on the same day or scheduled within one week and were conducted in private rooms within the clinic to maintain confidentiality. One patient refused participation and nineteen were unable to follow-up to complete the interview. The two interviewers included a research assistant and a physician who were trained by a US-based psychiatrist and epidemiologist in administration of all tools.

Vietnamese versions of the PHQ-9, GAD-7 and PC-PTSD-5 were administered by a research assistant. A separate physician interviewer, blinded to the results of the three screening tools, subsequently assessed each participant using the major depressive disorder, generalized anxiety disorder and PTSD sections of the MINI. Each participant answered a set of demographic, stigma and substance use questions in a background questionnaire that was verbally administered by the research assistant.

### Analyses

Total scores of the PHQ-9 (range: 0–27), GAD-7 (range: 0–21) and PC-PTSD-5 (range: 0–5), representing the number and frequency of endorsed symptoms, were compared with the reference gold standard MINI diagnoses using a receiver operating characteristic (ROC) curve [29]. An ROC curve graphs sensitivity vs specificity for all possible cut-off scores, and the area under the ROC curve (AUROC) is used to quantify the diagnostic ability of screening tool, where an AUC of 1 demonstrates perfect discrimination. The AUROC, sensitivities and specificities were calculated at each cut-off score for their respective screening tool [30]. The optimal cut-off score – the score that yielded the best sensitivity and specificity – was identified using the maximum value returned by Youden’s Index, a measure for optimizing the tradeoff between sensitivity and specificity [31].

Test characteristics (sensitivity, specificity and positive and negative likelihood ratios [LR+, LR-]) of the optimal PHQ-9, GAD-7, and PC-PTSD-5 scores were calculated relative to their respective diagnosis from the MINI [32]. All confidence intervals (CIs) were calculated with exact methods. Sensitivity and specificity were compared using Fisher exact test across strata of covariates. As the positive predictive values (PPV) and negative predictive values (NPV) are influenced by the prevalence of the disorder, which can have clinical implications, PPV and NPV were calculated for this population and for a range of hypothetical prevalence of depression and anxiety.

## Results

### Demographics

Of the 400 MMT patients enrolled, 99.3% were male (*n* = 397), 73% were married or partnered (*n* = 293), 48.5% had completed some high school (194) and 82.3% were working at least part-time (*n* = 329) (Table 1). A total of 21.8% (*n* = 87) were HIV positive, of which 95.4% (*n* = 83) were on antiretroviral therapy (ART).

**Table 1:** Characteristics of sample

Characteristic	Mean (SD)	n (%)
Overall		400 (100%)
Age	41.3 (7.2)	
Sex at birth		
Male		397 (99.3)
Female		3 (0.8)
HIV		
Negative		313 (78.25)
Positive		87 (21.75)
Marital Status		
Single		75 (18.75)
Married or partnered		293 (73.25)
Widowed/Divorced/Separated		32 (8)
Education		
None		2 (0.5)
At least some Primary		21 (5.25)
At least some Secondary		146 (36.5)
At least some High School		194 (48.5)
At least some Technical Training		7 (1.75)
At least some College		30 (7.5)
Employment		
Working at least part-time		329 (82.25)
Unemployed		64 (16)
Retired		7 (1.75)

### Prevalence

The prevalence of depression, generalized anxiety disorder and PTSD, respectively, was 10.5% (*n* = 42), 4% (*n* = 16) and 2% (*n* = 8), respectively, according to MINI criteria (Table 2). Approximately 11% of all patients had one or more CMD of which 3% (*n* = 12) had two disorders and 1.3% (*n* = 5) had all three disorders.

**Table 2:** Prevalence of Mental Health Disorders

Disorder	Mean (SD)	n (%)	Cronbach’s Alpha
Depression			
MINI Major Depressive Episode		42 (10.5)	
PHQ-9 Scores (range 0–27)	2.3 (4.3)		0.8803
Anxiety			
MINI Generalized Anxiety Disorder		16 (4.0)	
GAD7 Scores (range 0–21)	1.2 (2.9)		0.9111
PTSD			
MINI PTSD		8 (2.0)	
Screener Scores (range 0–6)	0.6 (1.2)		0.8426
Any CMD		44 (11.0)	
2 CMDs		12 (3.0)	
3 CMDs		5 (1.3)	

### Test characteristics

#### PHQ-9 screen validation against the MINI for major depressive disorder

**Fig. 1:**
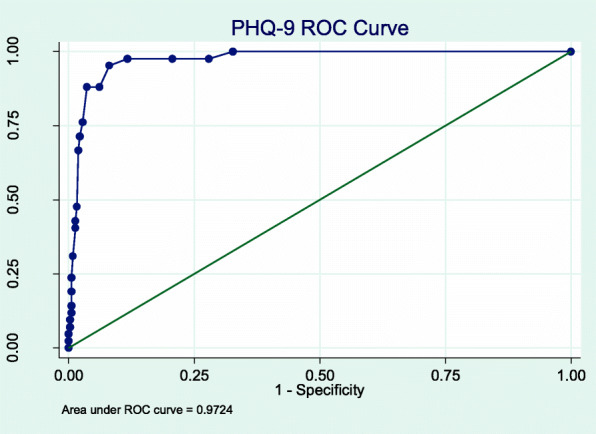
PHQ-9 ROC Curve

**Fig. 2:**
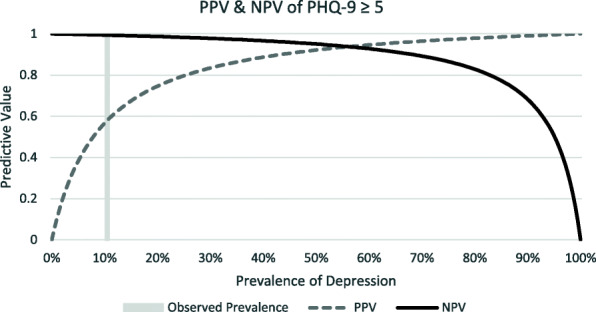
PHQ-9 PPV & NPV

A cut-off score of ≥5 gave a sensitivity of 95.2% (95% CI 83.9, 99.4) and a specificity of 91.9% (95% CI 88.6, 94.5) (Fig. 1). In this population with a prevalence of 10.5%, this correctly classified 92.3% of participants, yielding a PPV of 0.58 (95%CI 0.45, 0.7) and an NPV of 0.99 (95% CI 0.98, 1.00) (Fig. 2). The ROC analysis gave an AUC of 0.97 (CI 0.95–0.99) (Table 3). There were not significant differences between the sensitivity or specificity of the PHQ-9 among various sub-populations (Table 4).

**Table 3:** AUC, Cut-off Score, Sensitivity and Specificity by Screening Tool

Screening Tool	AUC (95%CI)	Cut-off Score	Sensitivity (95%CI)	Specificity (95%CI)	Correctly Classified	LR+	LR-
PHQ-9	0.97 (0.95, 0.99)	≥5	0.95 (0.84, 0.99)	0.92 (0.89, 0.95)	0.92	11.76	0.05
GAD-7	0.94 (0.87, 1.00)	≥3	0.94 (0.7, 1.00)	0.88 (0.84, 0.91)	0.95	15.31	0.39
PC-PTSD-5	0.86 (0.71, 1.00)	≥4	0.63 (0.24, 0.91)	0.96 (0.93, 0.98)	0.88	7.50	0.07

**Table 4:** Comparison of Test Characteristics of Screeners Between Subgroups

Characteristic	Depression (PHQ-9 Score > =10)					Anxiety (GAD-7)				
	Prevalence	Sensitivity	***P***-Value*	Specificity	***P***-value*	Prevalence	Sensitivity	***P***-Value*	Specificity	***P***-value*
Age			0.565		0.529			1.000		0.578
20–29	6.3	100		86.7		0.0	–		93.8	
30–39	11.3	100		94.0		5.3	87.5		90.1	
40–49	11.5	90.5		91.9		3.8	100.0		85.1	
50+	4.8	100		90.0		0.0	–		88.1	
Sex at birth			1		1			1.000		1.000
Male	10.3	95.1		0.0		3.8	93.3		87.4	
Female	33.3	100		100.0		33.3	100.0		100	
HIV			1		0.628			1.000		**0.013**
Negative	8.6	96.3		0.0		3.2	90.0		89.8	
Positive	17.2	93.3		90.3		6.9	100.0		79	
Marital Status			1		0.125			1.000		0.837
Single	9.3	100		88.2		4.0	100.0		86.1	
Married or partnered	10.9	93.8		93.5		4.1	91.7		87.9	
Widowed, Divorced, or Separated	9.4	100		86.2		3.1	100.0		87.1	
Education			0.408		0.161			0.500		0.066
< High School	9.1	100		93.5		3.7	100.0		90	
High School Diploma**	11.1	87.5		88.3		2.1	100.0		82.3	
College or beyond	16.2	100		96.8		13.5	80.0		93.8	
Employment			1		0.051			0.375		0.107
Working at least part-time	9.7	93.8		93.3		3.0	100.0		88.7	
Unemployed	15.6	100		83.3		9.4	83.3		79.3	
Retired	0	0		100.0		0.0	–		100	
MINI-Major Depressive Episode								0.125		**0.000**
No	–	–	–	–	–	0.6	50.0		92.7	
Yes	–	–	–	–	–	33.3	100.0		21.4	
MINI-Generalized Anxiety Disorder			0.545		0.156					
No	7.3	92.9		92.1		–	–	–	–	–
Yes	87.5	100		50.0		–	–	–	–	–

*Fisher exact test, 2 tailed

**Include technical training and college

#### GAD-7 validation against the MINI for generalized anxiety disorder

**Fig. 3:**
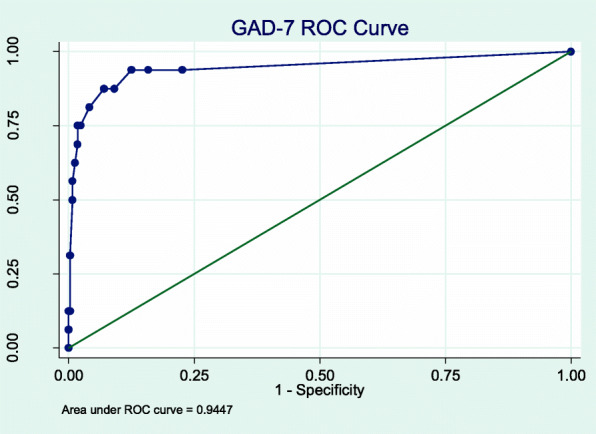
GAD-7 ROC Curve

**Fig. 4:**
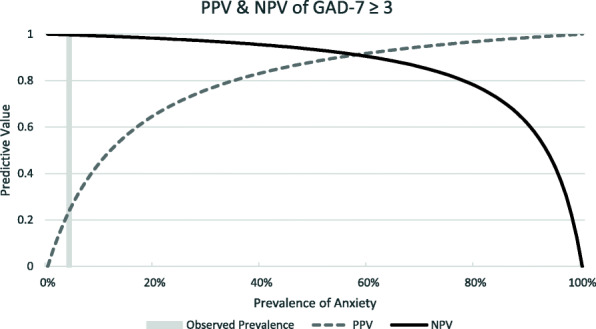
GAD-7 PPV & NPV

A cut-off score of ≥3 gave a sensitivity of 93.8% (95% CI 69.8, 99.8) and a specificity of 87.5% (95% CI 83.8, 90.6) (Fig. 3). In this population with a prevalence of 4.0%, this correctly classified 95.3% of participants, yielding a PPV of 0.24 (95% CI 0.14, 0.36) and an NPV of 1.00 (95% CI 0.98, 1.00) (Fig. 4). The ROC analysis gave an AUC of 0.94 (95% CI 0.87, 1.00) (Table 3). The GAD-7 was less specific for those living with HIV than those without (79.0% vs 89.8%, *P* = 0.013) and for those with major depressive disorder compared to those without (21.4% vs 92.7%, *P* < 0.001) (Table 4).

#### PC-PTSD-5 validation against the MINI for PTSD

**Fig. 5:**
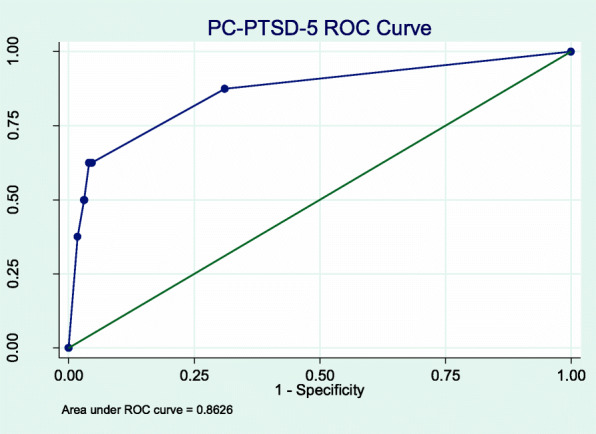
PC-PTSD-5 ROC Curve

**Fig. 6:**
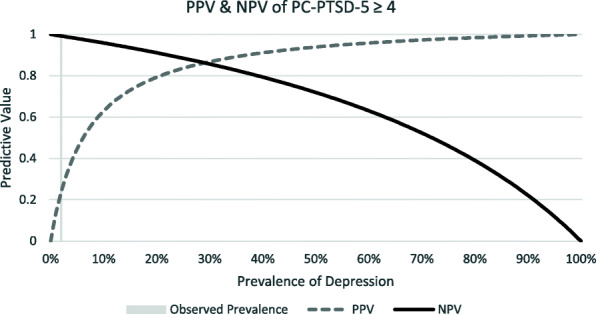
PC-PTSD-5 PPV & NPV

A cut-off score of ≥4 gave a sensitivity of 62.5% (95% CI 24.5, 91.5) and a specificity of 95.2% (95% CI 93.5, 97.6) (Fig. 5). In this population with a prevalence of 2.0%, this correctly classified 87.8% of participants, yielding a PPV of 0.24 (95% CI 0.08, 0.47) and an NPV of 0.99 (95% CI 0.98, 1.00) (Fig. 6). The ROC analysis gave an AUC of 0.86 (95% CI 0.71, 1.00) (Table 3). Due to the low prevalence of PTSD, we did not investigate differences in sensitivity and specificity among various sub-populations.

## Discussion

Our study aimed to validate tools to detect CMDs using the PHQ-9, GAD-7 and PC-PTSD-5 among the MMT population at an urban clinic in Hanoi. The screening tools validated in this study were developed in HICs and therefore required validation locally to determine appropriate cut-off scores, sensitivity, and specificity for the Vietnamese MMT population. The PHQ-9, GAD-7, and PC-PTSD-5 were selected to allow for individual screening of CMDs with separate tools and to our knowledge had not yet been validated in this population. We previously highlighted the importance of implementing well-designed studies that validate screening tools needed to detect CMDs [33]. Our validation of the PHQ-9, GAD-7 and PC-PTSD-5 are the first of their kind in the MMT population in Vietnam to our knowledge [34].

Using the MINI, we found that 11% of the participants had one or more CMDs while the prevalence of depression, anxiety and PTSD were 10.5, 4 and 2%, respectively. The prevalence of depression has been documented as high as 50% among methadone patients in HICs [15] with rates remaining consistently high within other countries in proximity to Vietnam such as China [35] (38%) and Malaysia [36] (44%) with the use of various screening tools. The literature suggests that overall CMD prevalence within Vietnam is lower than surrounding South East Asian countries, both within the general and MMT population. A study of MMT patients in northern Vietnam estimated a prevalence rate of 26.8% for any mental health pathology using the Kessler psychological distress scale [18]. Another study conducted among MMT patients in Vietnam using the DASS-21 estimated a 3.9% prevalence of mild to extremely severe depression and a 18% prevalence of mild to extremely severe anxiety [8]. Regional differences between CMD prevalence within Vietnam further complicate true rates. The majority of CMD studies in the MMT population have largely been limited to the northern rural provinces, documenting increased prevalence of anxiety or depression in rural (43.1%) as compared to urban areas (18.1%) [18, 19]. Prevalence of specific disorders remain lower in urban areas as seen for depression (12.2%) as identified using the MINI in Hai Phong for people who inject drugs [17]. Our findings remain higher than Vietnamese population estimates by the Ministry of Health for depression (2.8%) and anxiety (2.6%) [8].

A number of possible explanations for lower prevalence of CMDs in the MMT population exist. First, this rate may be due to a gender-biased sample, as 98% of participants were male. Globally, CMDs are more prevalent in females than males, especially anxiety disorders [2]. It is difficult to discern whether there are significantly more women who inject drugs who do not present for care as our male-predominant sample is consistent with similar studies of Vietnamese MMT patients [18]. Second, at MMT enrollment, patients are asked two questions to screen for severe mental health disorders with the potential for referral for psychiatric treatment. Among the study sample, up to 25 patients were referred for psychiatric services upon MMT initiation, which could have artificially decreased the prevalence of CMDs in our study. Third, the screening tools were developed using Western manifestations of CMDs according to the DSM-5 criteria; these criteria may not be as sensitive in detecting CMDs in the MMT population in Hanoi due to low prevalence rates and low cut-off scores for all three disorders and screening tools, respectively. CMDs may manifest with more somatic symptoms (such as headaches and neck pain, back pain, fatigue, and palpitations) among the Vietnamese population, as has been seen previously and in other LMICs [37]. Screening tools targeting somatic symptoms should be used in future screening tools to assess whether the prevalence of CMDs increases. Finally, cultural, household and stigma factors remain a leading explanation as to variable rates of CMD between MMT patients living in Vietnam [38].

The cut-off scores for two of the three screening tools tested were lower compared to respective cut-off scores in HICs. The PHQ-9 had an optimal cut-off score of ≥5 with a high sensitivity (95%) and high specificity (92%) compared to its validated HIC cut-off of 8–11, tiered according to severity [37]. The optimal cut-off score for the GAD-7 was ≥3 with a sensitivity of 94% and a specificity of 88%, one point below the HIC cut-off score for mild anxiety [25]. The optimal cut-off score for the PC-PTSD-5 Checklist was ≥4 with a sensitivity of 62.5% and specificity of 95.2%, which is the recommended cut-off score for further evaluation in HICs [23]. These findings have implications for clinical practice as lower cut-offs would prompt consideration of a confirming clinical assessment with fewer endorsed symptoms, meaning that clinicians needed to be more sensitive to the presence of a CMD than standard screen thresholds would suggest. Also in clinical practice, the PPV and NPV of these tools will depend on the prevalence of the CMDs. This population had a low prevalence of depression (10.5%), anxiety (4.0%) and PTSD (2%), resulting in NPVs near 100%, but lower PPVs. (Figs. 2, 4 and 6). In populations with higher CMD prevalence [35, 36], as previously documented in other studies discussed above, the NPVs will still be very high and the PPV will increase markedly. To our knowledge, there have been no validation studies of PTSD in the Vietnamese population.

Validation studies are critical for understanding how to apply a screening tool among different populations. However, the majority of CMD studies within Vietnam have focused on prevalence instead of validation [34]. Of the six studies validating mental health screening tools within Vietnam through 2014, none validated the PHQ-9, GAD-7 or any form of PTSD screening [34]. The SRQ-20 was validated in a district and community sample in rural Vietnam and reported lower cut-off scores than the WHO (World Health Organization) has previously recommended [39, 40]. Validations of the Zung SAS, EPDS and GHQ-12 among perinatal women in Vietnam similarly required lower cut-off scores [9]. Such findings alongside our own, highlight the utility of validation studies and suggest that screening tools for CMDs may not be accurately interpreted without prior validation.

Our study was limited to a single urban methadone clinic in Hanoi. We used translations of the PHQ-9 and GAD-7 from a previous study in a similar Vietnamese healthcare population [27]. The multi-step approach including independent comparison to a previously validated version; cultural applicability and comprehensibility; translation by an experienced, bilingual clinical staff person; and practice interviews is similar to methods from existing publications and our findings are consistent with those in other LMICs, minimizing lack of validation as a weakness [41–45]. While the cut-off score supported by our data is lower than standard cut-off scores in HICs, it is consistent with approaches used in other LMIC countries in non-psychiatric settings to indicate mild depression and other work indicating a lower threshold for major depressive disorder in Southeast Asian populations [41–46]. Our study included a large sample size with a high enrollment percentage (95% of clinic sampled) and blinding of the interviewers. We had a multidisciplinary team including both Vietnamese and American physicians, public health epidemiologists, lay health care workers and bilingual staff members to maximize adequate design of the study.

Our findings have several implications for future research. First, these findings need to be replicated to confirm their accuracy. Future validation studies could consider a mixed-methods approach to additionally investigate content validity of the measures. Further local adaptation and validation research could pilot screening tools that include more somatic symptoms or create novel screening tools that more purposefully capture local idioms and concepts of psychiatric distress. Samples should include more gender diversity when possible and include patients from multiple urban methadone clinics. Finally, in addition to establishing the accuracy of screening tools compared to a reference gold standard, implementation science research is needed to investigate the feasibility of integrating routine screening for CMDs, particularly in LMICs such as Vietnam, in order to close the research-to-practice gap in mental health care.

## Conclusion

The prevalence of one or more CMD among the MMT patient population was 11%. Prevalence of depression, anxiety and PTSD were 10.5, 4 and 2%, respectively. Prevalence of CMDs remained higher than the general Vietnamese population but may be lower than other reported prevalence of CMDs within the MMT population due to patients with severe mental illness having been referred for treatment prior to study engagement and other cultural factors. Optimal cut-off scores for the PHQ-9 and GAD-7 were lower than respective cut-off scores in HICs while the optimal cut-off score for PTSD remained the same.

## Data Availability

The datasets used and/or analyzed during the current study are available from the corresponding author on reasonable request.
